# The protective effect of sinapic acid in osteoarthritis: In vitro and in vivo studies

**DOI:** 10.1111/jcmm.14096

**Published:** 2019-01-03

**Authors:** Xiaobin Li, Jian Lin, Xiaoxia Ding, Jiangwei Xuan, Zhichao Hu, Dengying Wu, Xingyu Zhu, Zhenhua Feng, Wenfei Ni, Aimin Wu

**Affiliations:** ^1^ Department of Orthopaedic Surgery The Second Affiliated Hospital and Yuying Children’s Hospital of Wenzhou Medical University Wenzhou China; ^2^ Department of Chemoradiation Oncology The First Affiliated Hospital of Wenzhou Medical University Wenzhou China; ^3^ The Second Clinical Medical School of the Wenzhou Medical University Wenzhou China; ^4^ Department of Orthopaedic Surgery The Second Affiliated Hospital of Jiaxing University Jiaxing China

**Keywords:** chondrocytes, IL‐1β, inflammation, NF‐κB, Nrf2, sinapic acid

## Abstract

The anti‐inflammatory effect of sinapic acid (SA) has been reported in several studies. However, whether SA has the same effect on osteoarthritis (OA) has yet to be clearly elucidated. We designed a series of in vitro and in vivo procedures to verify the above conjecture. Compared with controls, SA‐pretreated human chondrocytes showed lower levels of interleukin (IL)‐1β‐induced IL‐6, prostaglandin E2 (PGE2), nitric oxide (NO) and tumour necrosis factor‐α (TNF‐α) in vitro. Meanwhile, SA could also reverse the degradation of type II collage and aggrecan, as well as the overproduction of matrix metalloproteinase‐9 (MMP‐9) and matrix metalloproteinase‐13 (MMP‐13), inducible nitric oxide synthase (iNOS), cyclooxygenase (COX)‐2 and a disintegrin and metalloproteinase thrombospondin motifs (ADAMTS)‐5. Furthermore, activation of nuclear factor κB (NF‐κB), which was induced by IL‐1β, was also inhibited by SA through the pathway of nuclear factor‐erythroid 2‐related factor‐2 (Nrf2)/heme oxygenase 1. In vivo, SA could delay the progress of mice OA models. We propose that SA may be applied as a potential therapeutic drug in OA treatment.

## 1 INTRODUCTION

Osteoarthritis (OA) is regarded as the most common form of chronic arthritis and is a painful degenerative joint diseasethat affects life quality, particularly, of elderly people.^1^ Progressive degradation of articular cartilage and synovial inflammation are the pathological features of OA.^2^ While the etiology of OA remains unclear, inflammation is considered to be involved in the pathogenesis of the disease.^3^ Inflammation cytokines including interleukin (IL)‐6, IL‐1β and tumor necrosis factor‐α (TNF‐α) are involved in the pathophysiology of OA.^4^ IL‐1β increases the production of a variety of pro‐inflammatory mediators, including prostaglandin E2 (PGE2), nitric oxide (NO), a disintegrin and metalloproteinase thrombospondin motifs (ADAMTS), a disintegrin and matrix metalloproteinases (MMPs) to promote the degradation of extracellular matrix (ECM).^5^, ^6^ Furthermore, accumulated evidence suggests that inhibition of the inflammation caused by IL‐1β may be a potential therapeutic target for patients with OA.^7^ It was well known that nuclear factor κB (NF‐κB) participated in the production of several inflammatory factors which were involved in the pathogenesis of OA and induced by IL‐1β.^8^, ^9^ As an important transcription factor in maintaining cell homeostasis, nuclear factor‐erythroid 2‐related factor‐2 (Nrf2) could interact with a variety of signalling pathways to exert antioxidant and anti‐inflammatory effects. An increasing amount of evidence demonstrates that there was an interaction between members of Nrf2 and NF‐κB pathways.^10^ Expression of antioxidant gene such as heme oxygenase‐1 (HO‐1), superoxide dismutase and NAD(P)H‐quinone oxidoreductase‐1 could be induced by the activation of Nrf2.^10^, ^11^ Among them, HO‐1 plays a pivotal role in Nrf2‐mediated NF‐κB inhibition.^10^ As reported, the production of inflammatory cytokines and NF‐κB activation could be inhibited by the activation of the Nrf2 signalling pathway.^12^, ^13^, ^14^ Furthermore, Nrf2 activation shows a chondroprotective potential in mice OA models.^15^ Targeting Nrf2 may therefore be useful in treating cartilage degeneration.

Sinapic acid (SA) is widely found in spices, fruits, vegetables, cereals and oilseed crops.^16^ It has been reported that SA has various biological functions such as antimicrobial, anti‐inflammatory, antioxidant as well as anti‐anxiety effects.^17^, ^18^ Previous studies discovered that SA could inhibit lipopolysaccharide‐induced inflammation through NF‐κB inactivation in vascular endothelial cells.^19^ Meanwhile, SA reduced the production of PGE2, IL‐1β, TNF‐α, inducible nitric oxide synthase (iNOS) and cyclooxygenase (COX)‐2 in RAW 264.7 macrophages.^20^ Additionally, SA activated the Nrf2/HO‐1 pathway, and led to NF‐κB inactivation in cisplatin‐induced nephrotoxicity in rats.^21^ However, the anti‐inflammatory effect of SA in OA has never been investigated. In this study, we used in vivo and in vitro methods to find out whether SA has an anti‐inflammatory effect of in OA and the possible underlying mechanism of SA.

## 2 MATERIALS AND METHODS

### 2.1 Reagents

Sinapic acid (purity ≥98%), carboxymethylcellulose, collagenase type II, recombinant human IL‐1β and dimethylsulfoxide (DMSO) were purchased from Sigma Chemical Co. (St. Louis, MO). SA was dissolved in 0.5% carboxymethylcellulose sodium to form oral suspension. Cell‐counting kit‐8 (CCK‐8) was obtained from Dojindo (Kumamo, Japan). Primary antibodies against COX‐2 and iNOS were purchased from Abcam (Cambridge, MA). Primary antibodies against p65, inhibitor of kappa B (IκB)‐α, Nrf2 and HO‐1 were acquired from CST (Danvers, MA). TRIzol reagent was obtained from Invitrogen (Carlsbad, CA). Griess reagent was acquired from Beyotime Institute of Biotechnology (Shanghai, China). SYBR Green Master Mix was purchased from Bio‐Rad Laboratories (Hercules, CA). QuantiTect Reverse Transcription kit was purchased from Qiagen (Valencia, CA). ELISA kits of PGE2, TNF‐α, IL‐6, MMP‐13 and ADAMTS‐5 were purchased from R&D systems (Minneapolis, MN).

### 2.2 Isolation and culture of chondrocytes

Tissue collection was according to the terms of the Medical Ethical Committee of the Second Affiliated Hospital, Wenzhou Medical University (ethic cord: LCKY‐2017‐30) and following the guidelines of the Declaration of Helsinki. Human cartilage samples were acquired from OA patients (aged 52‐64 years, full ethical consent was obtained) underwent total knee replacement surgery at the Second Affiliated Hospital of Wenzhou Medical University. All the patients’ knee cartilage tissues were collected under aseptic conditions. To avoid other cells contamination, cartilage tissues were separated from underlying bone and connective tissues. Afterwards, cartilage tissues were cut into small pieces and then digested with a 0.25% trypsin‐EDTA solution for 30 minutes and 0.1% collagenase II in DMEM/F12 at 37°C for 4 hours. Liquid supernatant was collected and then centrifuged at 100 *g* for 5 minutes. The inner cell mass was obtained and suspended in DMEM/F12 with 10% FBS and 1% antibiotic mixture and incubated in an atmosphere of 95% air and 5% CO_2_ at 37°C. The medium was changed every 2‐3 days. Cells were passaged when up to 80% to 90% confluence using 0.25% trypsin‐EDTA solution. To avoid the phenotype loss, only passage 1 and 2 were used in our study.

### 2.3 CCK‐8 assay

In all cell experiments, the concentration of DMSO was <1‰. The effects of SA on cell viability on human chondrocytes were determined using a CCK‐8 kit according to the manufacturer's instructions. Briefly, human OA chondrocytes were seeded onto 96‐well plates (6000/well) to adherence for 12 hours and then incubated in various concentrations (40, 80, 160 and 320 μmol/L) Of SA for 24 hours. Later, 10 μL of CCK‐8 was added to each well and incubated at 37°C for another 4 hours. The absorbance of the wells at 450 nm was then measured using a microplate reader (Leica Microsystems, Wetzlar, Germany).

### 2.4 Griess reaction and ELISAs

The interaction of NO in culture medium was determined by Griess reaction as previously described, while the concentration of PGE2, MMP‐1, MMP‐13 and ADAMTS‐5 in the culture medium was detected using commercial ELISA kit (Minneapolis, MN) according to the manufacturer's instruction. All assays were performed in duplicate.

### 2.5 qRT‐PCR

Total RNA of human chondrocytes incubated with various concentration of SA were extracted using TRIzol reagent according to the manufacturer's instructions and then the concentration was determined spectrophotometrically at 260 nm (Thermo Scientific NanoDrop 2000) (Middlesex,MA). The quality and purity was verified using A260/A280 ratio. A total of 1000 ng of total RNA was reverse transcribed to synthesize cDNA (MBI Fermantas, Mooney, Germany). Quantitative real‐time PCR (qPCR) was performed using CFX96Real‐TimePCR System (Bio‐Rad Laboratories). Parameters of RT‐PCR were: 10 minutes 95°C, followed by 40 cycles of 15 seconds, 95°C and 1 minute 60°C. The reaction was performed in a total volume of 10 μL, concluding 4.5 μL diluted cDNA, 4.5 μL SYBR Green Master Mix, 0.25 μL forward primer and 0.25 μL reverse primer. The level of target mRNA was normalized to the level of GADPH. The level of relative mRNA of each target gene was analysed using 2^−ΔΔCT ^method. Each gene analysis was performed in triplicate. Primer's sequences of the targeted genes were designed with the aid of NCBI Primer‐Blast Tool and listed in Table 1.

**Table 1:** Primer sequences used in qRT‐PCR

Gene	Forward primer	Reverse primer
COX‐2	5′‐GAGAGATGTATCCTCCCACAGTCA‐3′	5′‐GACCAGGCACCAGACCAAAG‐3′
iNOS	5′‐CCTTACGAGGCGAAGAAGGACAG‐3′	5′‐CAGTTTGAGAGAGGAGGCTCCG‐3′
TNF‐α	5′‐GTCAGATCATCTTCTCGAACC‐3′	5′‐CAGATAGATGGGCTCATACC‐3′
IL‐6	5′‐GACAGCCACTCACCTCTTCA‐3′	5′‐TTCACCAGGCAAGTCTCCTC‐3′
MMP‐13	5′‐CCAGAACTTCCCAACCAT‐3′	5′‐ACCCTCCATAATGTCATACC‐3′
ADAMTS‐5	5′‐GCAGAACATCGACCAACTCTACTC‐3′	5′‐CCAGCAATGCCCACCGAAC‐3′
Collagen‐II	5′‐CTCAAGTCGCTGAACAACCA‐3′	5′‐GTCTCCGCTCTTCCACTCTG‐3′
Aggrecan	5′‐AAGTGCTATGCTGGCTGGTT‐3′	5′‐GGTCTGGTTGGGGTAGAGGT‐3′
GADPH	5′‐TCTCCTCTGACTTCAACAGCGAC‐3′	5′‐CCCTGTTGCTGTAGCCAAATTC‐3′

ADAMTS, a disintegrin and metalloproteinase thrombospondin motifs; COX‐2, cyclooxygenase; IL, interleukin; iNOS, inducible nitric oxide synthase; MMP, matrix metalloproteinase; TNF‐α, tumour necrosis factor‐α.

### 2.6 Western blotting

Total proteins were extracted from human chondrocytes using RIPA lysis buffer with 1 mmol/L Phenylmethanesulfonyl fluoride and on the ice for 10 minutes followed by 15 minutes centrifugation at 13 800 *g* and 4°C, and then protein concentration was measured using the BCA protein assay kit. Forty micrograms of protein were loaded onto an SDS‐PAGE gel and transferred to a PVDF membrane (Bio‐Rad). The membranes were blocked with 5% non‐fat dry milk for 2 hours at room temperature and subsequently incubated sequentially with primary antibodies(all 1:1000 dilution) against p65, IκB‐α, iNOs, COX‐2, Nrf2, HO‐1, MMP‐9, MMP‐13, Lamin‐B and GADPH overnight at 4°C, and followed by subsequently incubation in HRP‐conjugated secondary antibodies (1:3000) for 2 hours at room temperature. After washing three times with TBST for 5 minutes, the blots were visualized by electrochemiluminescence plus reagent (Invitrogen) and afterwards the intensity of these membranes was quantified with Image Lab 3.0 software (Bio‐Rad).

### 2.7 siRNA transfection

Double‐stranded siRNA for human Nrf2 gene silencing was designed and chemically synthesized (RiboBio, Guangzhou, China) and the sequences of the Nrf2 siRNA was as follows: sense strand 5′‐GUAAGAAGCCAGAUGUUAADUDU‐3′. Nrf2 and negative control of siRNA transfection were undertaken using Lipofectamine 2000 siRNA transfection reagent (Thermo Fisher, UT, USA).

### 2.8 Immunofluorescence microscopy

Human chondrocytes were seeded on slices in glass plates in a six‐well plate at a density of 3 × 10^5^ cells/mL and incubated for 24 hours. Afterwards, glass coverslips with chondrocyte monolayers were rinsed three times in PBS before fixation using 4% paraformaldehyde for 15 minutes and followed by permeation using the 0.5% Triton X‐100 for 15 minutes at room temperature. Then, the sample was blocked with 5% goat serum for 1 hour at room temperature, rinsed with PBS and incubated with primary antibody against collagen‐II and p65 (1:200) at 4°C overnight. On the next day, the glass plates were incubated with fluorescein‐conjugated goat anti‐rabbit IgG antibody (1:400) for 1 hour and labelled with DAPI (Invitrogen) for 1 minute. Finally, images were captured using a fluorescence microscope (Olympus Inc, Tokyo, Japan).

### 2.9 Animal experiments

Ten‐week‐old C57BL/6 male wild‐type mice were purchased from the Animal Center of Chinese Academy of Sciences, Shanghai, China. The protocol for animal care and use conformed to the guidelines set forth by the Chinese National Institutes of Health and was approved by the Animal Care and Use Committee of Wenzhou Medical University. OA induction was performed as previously described. Briefly, after anaesthesia with peritoneal injection of 2% pentobarbital, the cranial attachment of the medial meniscus to the tibial plateau (medial meniscotibial ligament) of the right knee was transected with a microsurgical knife. The lateral meniscotibial ligament was identified and protected during the surgery. A sham operation, consisting of an arthrotomy without the transaction of medial meniscotibial ligament, was also performed in the right knee joint of mice in sham group.

In this study, mice were randomly divided into three groups of 10 mice, including a sham control group (sham), an OA group and an OA treated with SA group (SA). Mice in SA group received a gavage of SA (20 mg/kg/d) for 14 days after surgery while mice in OA group received a gavage of 0.5% carboxymethylcellulose sodium. All animals were sacrificed at 8 weeks after surgery. Knee joint tissues were collected for further analysis.

### 2.10 Histological analysis and immunohistochemistry

Knee joints were fixed in 4% paraformaldehyde for 24 hours and then decalcified in 10% EDTA solution for 1 month. The specimens then were embedded in paraffin and cut into 5 μm thick sections. The sections were stained with hematoxylin and eosin (H&E), Safranin‐O‐Fast green (S‐O) staining and Toluidine Blue staining. The histological assessment was performed according to the grading of Osteoarthritis Research Society International (OARSI) scoring system in a blinded manner as described previously. For immunohistochemistry*, *mouse cartilage sections (5 μm) were prepared using a freezing microtome and then the sections were overnight incubated at 4°C with primary antibodies for Nrf2, collagen‐II and MMP‐13. Afterwards, the histological sections were incubated with secondary antibodies (Beyotime Institute of Biotechnology Inc, Jiangsu, China) for 2 hours at room temperature. The DAB substrate system (Zsbio, Beijing, China) was used for colour development. Haematoxylin staining was utilized to reveal the nuclei of the cells. The number of positively stained cells on the entire articular surface per specimen was counted and the percentage positive cells was calculated.

### 2.11 Statistical analysis

Data were expressed as means ± standard deviation of the representative experiment performed in triplicate. Statistical analyses were performed using SPSS statistical software program 20.0. (Chicago, IL, USA） *P* < 0.05 was considered statistically significant.

## 3 RESULTS

### 3.1 Viability of human OA chondrocytes at different SA concentrations

**Figure 1:**
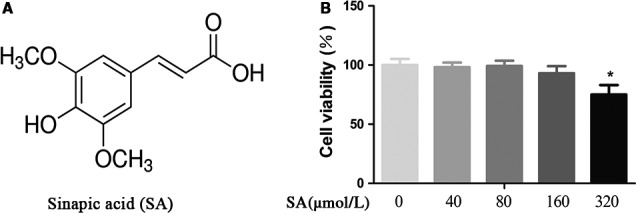
Effect of SA on human OA chondrocyte viability. A, Chemical structure of SA. B, Cells were treated with different concentrations of SA (40, 80, 160, and 320 μmol/L) for 24 hours and analysed using a CCK‐8 assay. Data are expressed as mean ± SD. All experiments were repeated three times. *P < 0.05 compared with control group. CCK‐8, cell‐counting kit‐8; OA, osteoarthritis; SA, sinapic acid

The chemical structure of SA (4‐hydroxy‐3,5‐dimethoxy cinnamic acid) was shown in Figure 1A. The viability of chondrocytes assessed by the cell counting kit‐8 assay at different SA concentrations assessed was shown in Figure 1B. The viability of chondrocytes was unaffected when concentration of SA was 40, 80 or 160 μmol/L. However, SA did significantly reduce cell viability at a concentration of 320 μmol/L. Thus, we chose SA at concentrations of 40, 80 and 160 μmol/L for further investigation.

### 3.2 SA inhibited the production of COX‐2, iNOS, PGE2, NO, IL‐6 and TNF‐α in human OA chondrocytes

**Figure 2:**
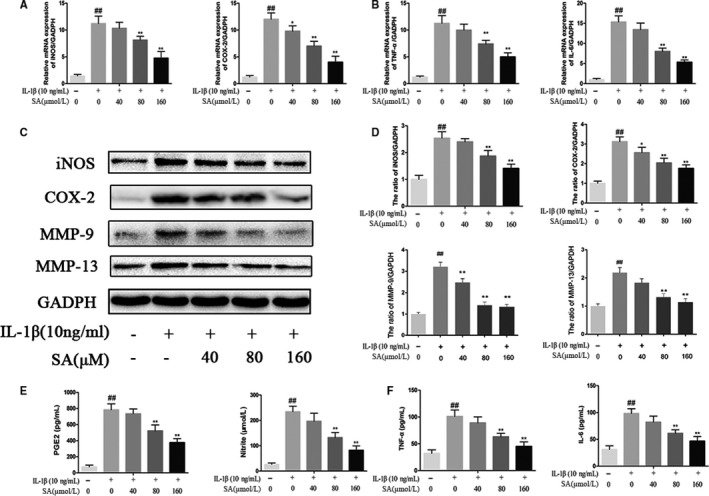
Effect of SA on IL‐1β‐induced inflammatory in human OA chondrocytes. Chondrocytes were pre‐treated for 1 hour with various concentrations of SA (40, 80, and 160 μmol/L) and then stimulated or without stimulated with IL‐1β (10 ng/mL) for 24 hours. The mRNA expression levels of iNOS, COX‐2, TNF‐α and IL‐6 were assayed by qRT‐PCR (A, B). The protein expression of iNOS, COX‐2, MMP‐9 and MMP‐13 assessed by Western blot and quantification analysis (C, D). Effect of SA on IL‐1β‐induced PGE2, NO, TNF‐α and IL‐6 production in human chondrocytes (E, F). Data are expressed as mean ± SD. All experiments were repeated three times. ^##^ *P* < 0.01 compared with control group. *P < 0.05, **P < 0.01 compared with IL‐1β group. COX‐2, cyclooxygenase‐2; IL, interleukin; iNOS, inducible nitric oxide synthase; MMP, matrix metalloproteinase; NO, nitric oxide; OA, osteoarthritis; PGE2, prostaglandin E2; SA, sinapic acid; TNF‐α, tumor necrosis factor‐α

To investigate the effect of SA on IL‐1β‐induced COX‐2 and iNOS expression in human chondrocytes, we performed Western blot and reverse transcription polymerase. Compared with control group, IL‐1β considerably upregulated COX‐2 and iNOS mRNA expression in human OA chondrocytes (Figure 2A). However, SA pre‐treatment inhibited the IL‐1β‐induced upregulation of COX‐2 and iNOS mRNA in a dose‐dependent manner (Figure 2A). Furthermore, similar results were found at the protein level of COX‐2 and iNOS expression (Figure 2C,D) and SA inhibited IL‐1β‐induced PGE2 and NO production dose‐dependently (Figure 2E). Additionally, the results of ELISA and RT‐PCR indicated that the inhibition of IL‐6 and TNF‐α production was concentration‐dependent (Figure 2B,F).

### 3.3 Effect of SA on ECM synthesis and degradation in IL‐1β‐induced human OA chondrocytes

**Figure 3:**
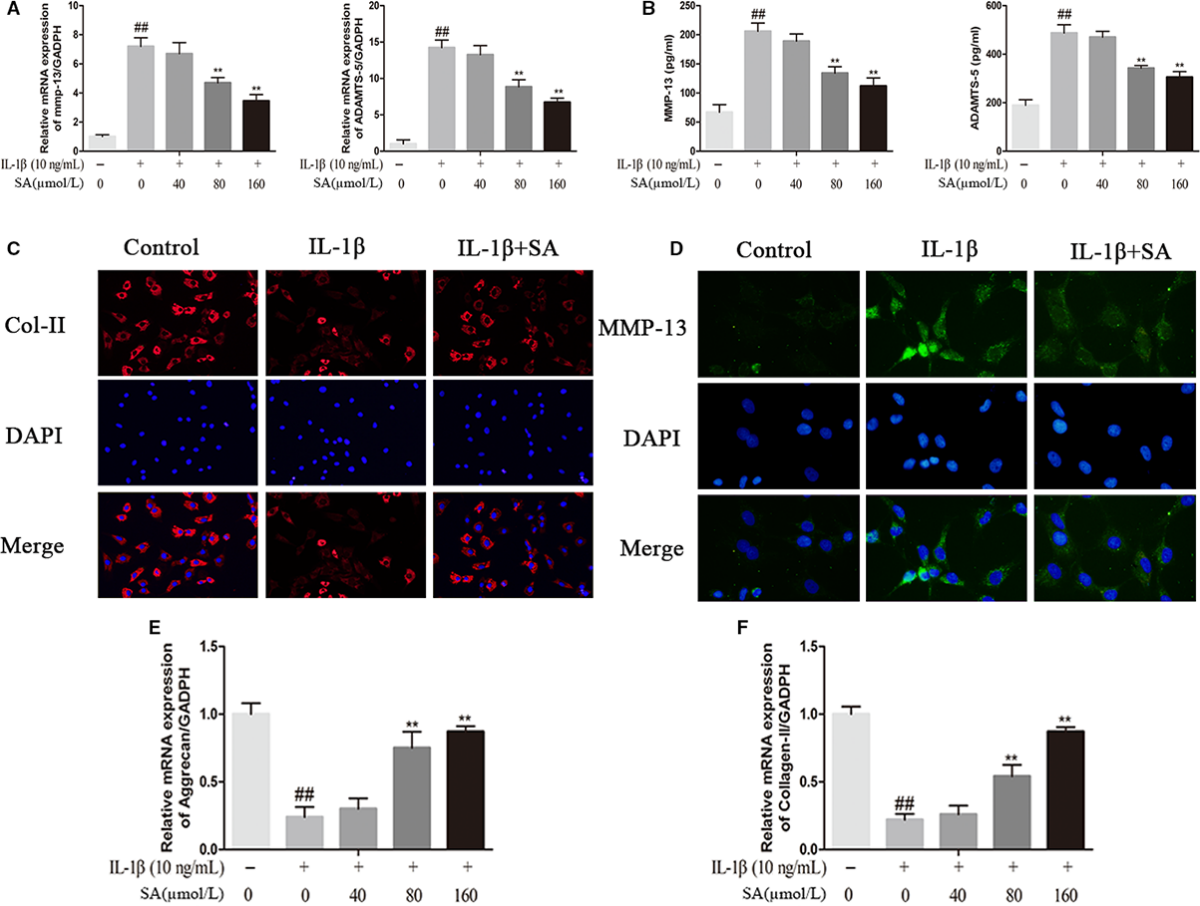
Effect of SA on extracellular matrix degradation from IL‐1β‐induced human OA chondrocytes. Chondrocytes were pre‐treated for 1 hour with various concentrations of SA (40, 80, and 160 μmol/L), followed by stimulation with or without IL‐1β (10 ng/mL) for 24 hours. The mRNA expression levels of MMP‐13, ADAMTS‐5 (A), aggrecan (E) and collagen‐II (F) were assayed by qRT‐PCR. The protein expression levels of MMP‐13, ADAMTS‐5 were determined by ELISA (B). The protein expression levels of collagen‐II (C) and MMP‐13 (D) were determined by immunofluorescence. The values are mean ± SD of three independent experiments. ^##^ *P* < 0.01 compared with control group. **P < 0.01 compared with IL‐1β group. ADAMTS, a disintegrin and metalloproteinase thrombospondin motifs; IL, interleukin; MMP, matrix metalloproteinase; OA, osteoarthritis; SA, sinapic acid

We pre‐treated chondrocytes with SA (40, 80, and 160 μmol/L) for 60 minutes and then chondrocytes were treated or untreated with IL‐1β (10 ng/mL) for 24 hours. The results showed that IL‐1β noticeably enhanced the expression of ADAMTS and MMP‐13 at mRNA and protein level, MMP‐9 at protein level in chondrocytes (Figure 2C,D), which were all remarkably decreased after SA treatment (Figure 3A). Otherwise, as shown in Figure 3E and F, SA distinctly reduced mRNA downregulation of type II collagen (collagen‐II) and aggrecan caused by IL‐1β stimulation. Consistently, immunofluorescence results revealed that SA considerably inhibited the protein degradation of collagen‐II and the expression of MMP‐13 (Figure 3C,D).

### 3.4 Effect of SA on NF‐κB activation in IL‐1β‐induced human OA chondrocytes

**Figure 4:**
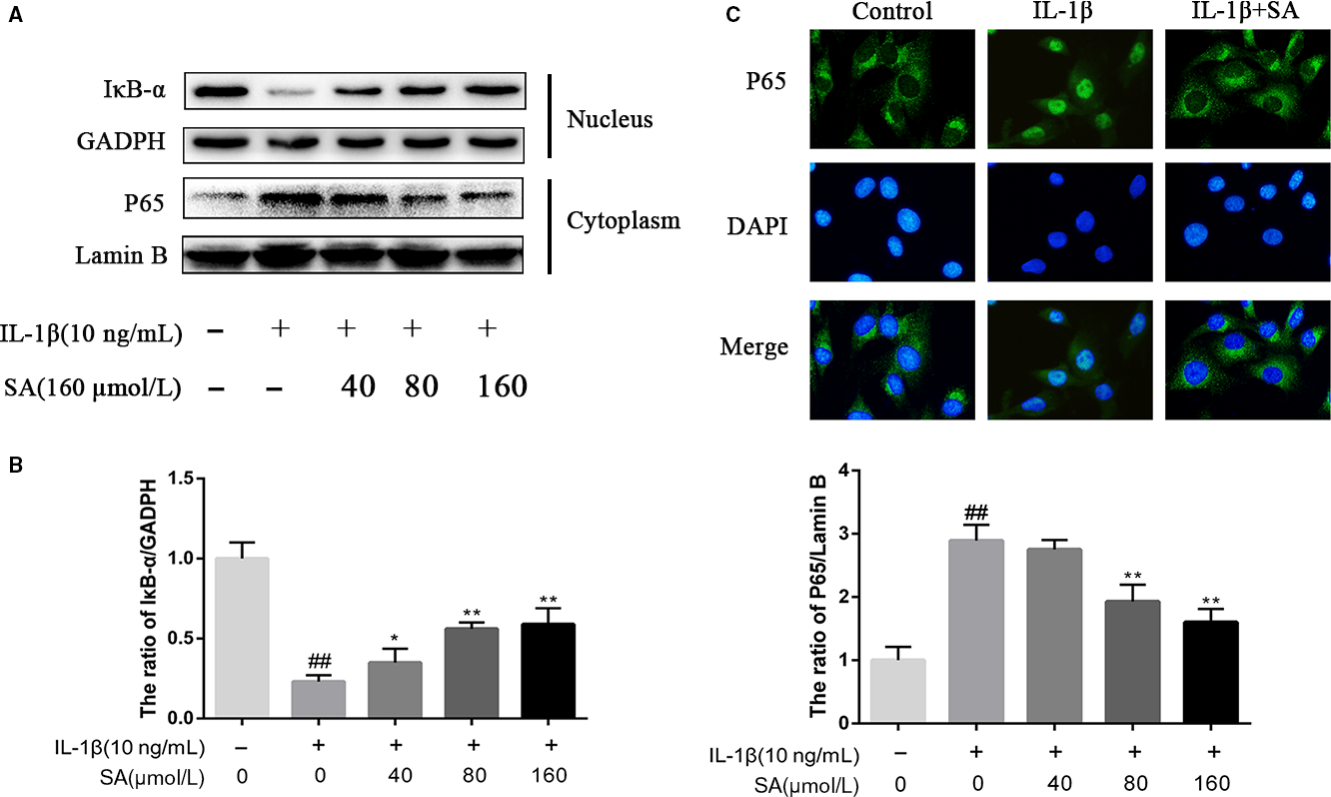
Effect of SA on IL‐1β‐induced NF‐κB activation in human OA chondrocytes. Chondrocytes were pre‐treated for 1 hour with various concentrations of SA (40, 80, and 160 μmol/L), followed by stimulation with or without IL‐1β (10 ng/mL) for 2 hours. NF‐κB p65 activation (in nucleus) and IκB‐α degradation (in cytoplasm) were determined by Western blot (A) and quantification analysis (B). The nuclei translocation of p65 was detected by immunofluorescence combined with DAPI staining for nuclei (C). The values are mean ± SD of three independent experiments. ^##^ *P* < 0.01 compared with control group. *P < 0.05, **P < 0.01 compared with IL‐1β group. IL, interleukin; IκB, inhibitor of kappa B; NF‐κB, nuclear factor κB; OA, osteoarthritis; SA, sinapic acid

IL‐1β stimulation led to the degradation of IκBα, and increasing phosphorylation of NF‐κB p65 and the IκB‐α in human OA chondrocytes, which could be dramatically inhibited by SA (Figure 4A). In order to determine whether SA could inhibit NF‐κB p65 nuclear translocation induced by IL‐1β, chondrocytes were pre‐treated with or without SA (160 μmol/L) for 60 minutes and followed by a stimulation with IL‐1β (10 ng/mL) for 120 minutes, after which immunofluorescence staining was performed. As shown in Figure 4C, SA could significantly inhibit the nuclear translocation of p65 induced by IL‐1β.

### 3.5 Effect of SA on the Nrf2/HO‐1 pathway in IL‐1β‐induced human OA chondrocytes

**Figure 5:**
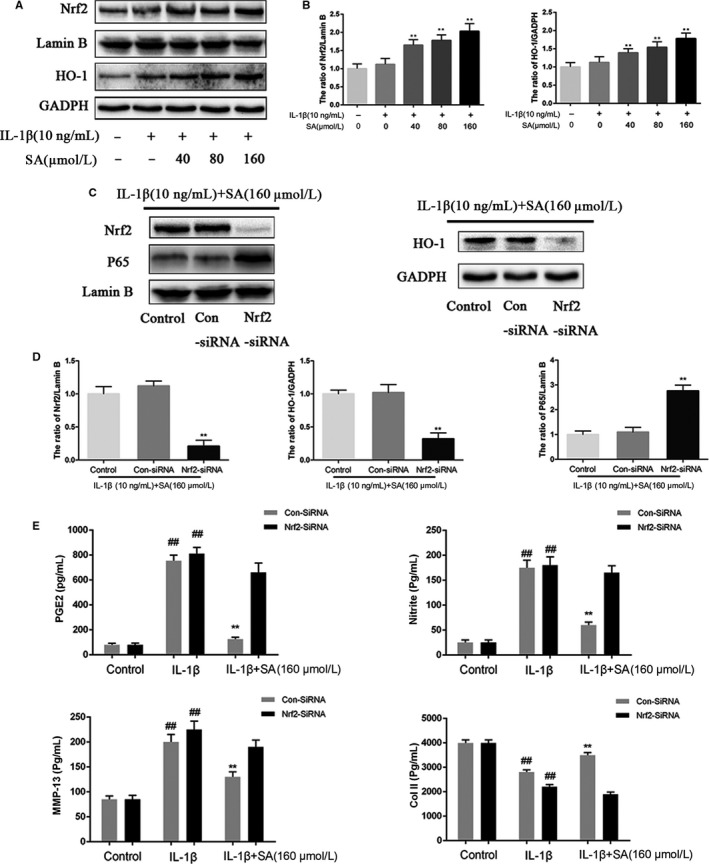
Effect of SA on Nrf2/HO‐1 pathway. Chondrocytes were pre‐treated for 1 hour with various concentrations of SA (40, 80, and 160 μmol/L), followed by stimulation with or without IL‐1β (10 ng/mL) for 2 hours. Nrf2 activation (in nucleus) and HO‐1 (in cytoplasm) were determined by Western blot (A) and quantification analysis (B). After Nrf2 knock down, the protein expressions of Nrf2 and p65 in nuclear and HO‐1 in cytoplasm in chondrocytes treated as above were visualized by Western blot (C), and quantified in (D). The production of PGE2, MMP‐13 and collagen‐ll was assessed by ELISA, and the expression of NO was assessed using Griess reagent (E). The values are mean ± SD of three independent experiments. ^##^P < 0.01 compared with Control group. **P < 0.01 compared with IL‐1β group. HO‐1, heme oxygenase‐1; IL, interleukin; Nrf2, nuclear factor‐erythroid 2‐related factor‐2; SA, sinapic acid; MMP, matrix metalloproteinase; NO, nitric oxide; PGE2, prostaglandin E2

IL‐1β stimulation had no effect on Nrf2 and HO‐1 expression, while pre‐treat chondrocytes with SA significantly enhanced their expression in a dose‐dependent manner (Figure 5A,B). Moreover, our results demonstrated that Nrf2 small interfering RNA (siRNA) transfection significantly inhibited cytoplasmic HO‐1 and nuclear Nrf2 expression in SA‐pre‐treated plus IL‐1β‐stimulated chondrocytes (Figure 5C,D). Furthermore, expression of nuclear p65 was considerably elevated after Nrf2 siRNA transfection, which suggested that the Nrf2 pathway mediates the anti‐inflammatory effect of SA in chondrocytes.

### 3.6 SA slowed the progression of OA in mice OA models

**Figure 6:**
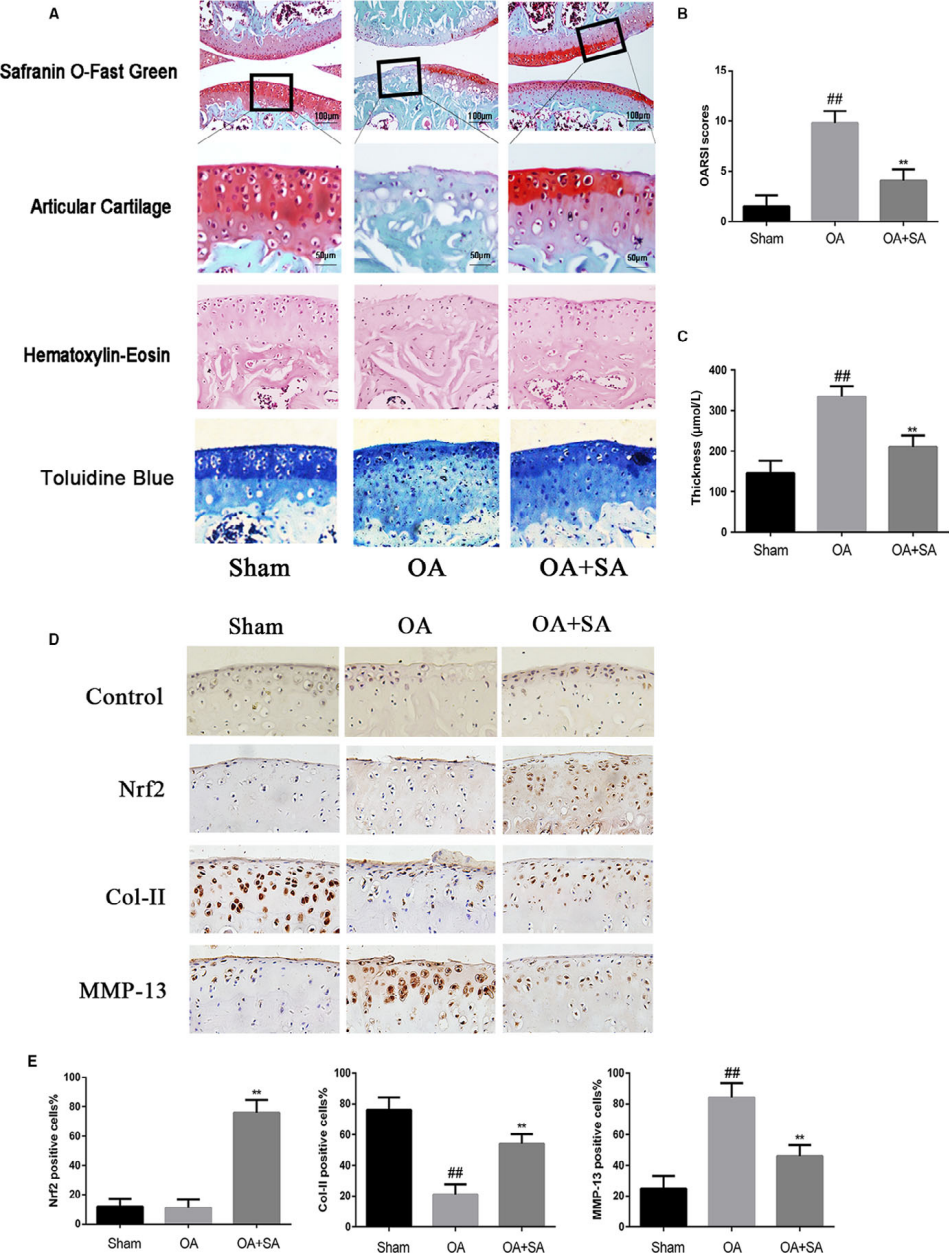
SA alleviated the progression of OA in mice OA models. Mice received a gavage of SA (20 mg/kg) or vehicle (carboxymethylcellulose sodium) 14 days after surgery. Histological analysis of OA was evaluated by Safranin O staining, Haematoxylin‐Eosin and Toluidine Blue staining (A). Osteoarthritis Research Society International (OARSI) scores (B) and subchondral bone plate thickness (C) were calculated for each group. Immunohistochemistry of Nrf2, collagen‐II and MMP‐13 were assessed to the effect of SA on cartilage matrix degradation in mice OA models. Quantifcation of Nrf2, collagen‐II and MMP‐13‐positive cells in cartilage samples (D, E). Data are mean ± SD. ^##^ *P* < 0.5 compared with control group, **P < 0.05 compared with OA group. n = 10 for each group. Representative histologic images are shown. Nrf2, nuclear factor‐erythroid 2‐related factor‐2; OA, osteoarthritis; SA, sinapic acid

We established OA model in mice by surgery. Then, either 20 mg/kg SA or a vehicle (carboxymethylcellulose sodium) were applied daily via gavage for 14 days. Histological analysis was assessed by OARSI scores in a blinded fashion by the treatment‐blind observers, subchondral bone plate thickness and Safranin O and H&E staining. Safranin O, Toluidine Blue and H&E staining results showed that, compared with OA group, the pathological manifestations such as superficial cartilage destruction, cartilage erosion, proteoglycan loss and hypocellularity were reduced in SA group (Figure 6). Besides, SA group also had a lower OARSI score and subchondral bone plate thickness, suggesting that SA could suppress the observed degradation of cartilage as well as slow the progression of OA.

### 3.7 Effect of SA on Nrf2, collagen‐II and MMP‐13 expression in OA articular cartilage

We performed immunohistochemistry to determine the expression of Nrf2, collagen‐II and MMP‐13 in cartilage matrix (Figure 6D). Quantitative analysis showed that treatment with SA significantly increased the number of positive Nrf2 cells (Figure 6D). Meanwhile, compared with the sham control group, the OA group showed a reduced number of collagen‐II and aggrecan positive cells, and an increased number of MMP‐13 positive cells. However, treatment with SA greatly increased the number of collagen‐II positive cells and decreased the number of MMP‐13 positive cells (Figure 6E).

## 4 DISCUSSION

The incidence of OA has increased alongside an increasingly ageing population.^22^ There is an increasing evidences that inflammation plays a critical role in the initiation and progression of OA.^23^ Currently, plant‐derived compounds are receiving increasing interest in the treatment of OA due to their minor side effects and anti‐inflammatory properties.^24^, ^25^ It has been demonstrated that SA, a plant‐derived compound, has a potential anti‐inflammatory property.^20^ In this study, we aimed to investigate whether SA has an anti‐inflammatory effect in chondrocytes and OA mice models. The results revealed that, in chondrocytes, SA exerts anti‐inflammatory effects via activation of the Nrf2 signalling pathway. Moreover, treatment with SA attenuated development of OA models.

As been reported, articular cartilage destruction as well as cartilage matrix degradation played significant role during the pathophysiology of OA, which could be promoted by inflammatory cytokine IL‐1β.^26^ Excessive delivery of PGE2 and NO is closely correlated with the pathophysiology of OA.^27^ A large amount of evidence has demonstrated that decreased production of inflammatory cytokines including NO and PGE2 could attenuate the progression of OA, as NO and PGE2 could inhibit ECM synthesis and induce ECM degradation.^28^, ^29^ Consistent with the results of Yun et al, we found that iNOS, COX‐2 and NO and PGE2 expression were strongly blocked by SA at both mRNA and protein levels induced by IL‐1β in human OA chondrocytes.^20^

MMPs, the most important proteolytic system in degrading the ECM, comprise a class of proteolytic enzymes. As reported in several studies, MMP‐9 and MMP‐13 could irreversibly and efficiently digest collagen‐II.^30^ ADAMTS‐5 plays the key role in the digestion of aggrecan.^31^ Growing evidence has demonstrated that in clinical patients, the progression of OA could be delayed by ADAMTS inhibitors,^32^ especially ADAMTS‐5 inhibitors, which lessened aggrecan loss in human OA cartilage explants through siRNA.^33^ In our research, we discovered that SA clearly reduced IL‐1β‐induced MMP‐13 and ADAMTS‐5 production in human OA chondrocytes at mRNA as well as protein levels.

It is well known that NF‐κB signalling pathway could regulator inflammatory mediators associated with OA development.^9^ As reported previously, phosphorylation of IκBα and p65 release could be triggered by IL‐1β stimulation, consequently translocated from the cytoplasm to the nucleus and lead to the inflammatory mediators production.^34^ In addition, NF‐κB p65‐specific siRNA activation could lead to the inhibition of NF‐κB p65 and COX‐2, iNOS, MMP‐9 and MMP‐13 production induced by IL‐1β in human chondrocytes. Furthermore, the Nrf2/HO‐1 pathway is deemed a target for inflammation induced by anti‐NF‐κB.^10^ To maintain its stability, Nrf2 normally binds to Kelch‐like ECH‐associated protein‐1 (Keap1) under normal physiological conditions. Nrf2, dissociating from Keap1, enters the nucleus and later unite with antioxidant‐responsive elements, resulting in the downstream genes upregulation, such as HO‐1. It has been reported that Nrf2 and HO‐1 could lighten inflammation through the inhibition of p65 translocation.^10^ It has also been reported that Nrf2 activation could prevent OA progress via inflammatory response inhibition.^15^ Moreover, SA led to the inhibition of NF‐κB in cisplatin‐induced nephrotoxicity in rats via activation of the Nrf2/HO1 pathway.^21^ In our results, SA inhibited the IL‐1β‐induced inflammatory response via the activation of Nrf2.

**Figure 7:**
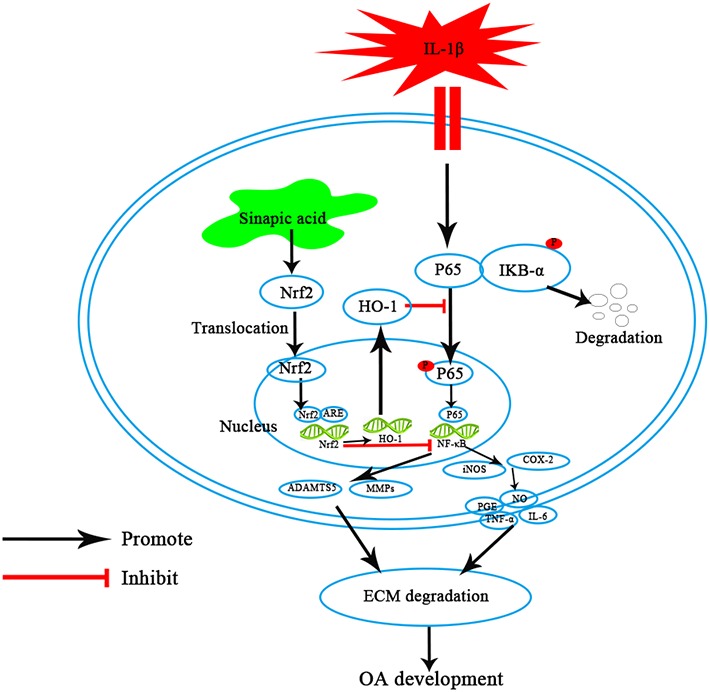
Working model for the inhibitory of SA on IL‐1β‐induced inflammation and ECM degradation in human OA chondrocytes by targeting the Nrf2/HO‐1/NF‐κB axis. ECM, extracellular matrix; HO‐1, heme oxygenase‐1; IL, interleukin; NF‐κB, nuclear factor κB; Nrf2, nuclear factor‐erythroid 2‐related factor‐2; OA, osteoarthritis; SA, sinapic acid

In conclusion, our findings demonstrated that SA could suppress the expression of IL‐1β‐induced inflammatory mediators by targeting the Nrf2/HO‐1/NF‐κB axis (Figure 7). Meanwhile, SA could reduce the degradation of ECM. Moreover, treatment with SA could decrease the OARSI scores and subchondral bone plate thickness in mice OA models, suggesting that SA may serve as a potential anti‐inflammatory agent in OA treatment. Further studies are needed to investigate the underlying mechanisms and clinical efficacy of SA on OA in more detail.

## CONFLICT OF INTEREST

The authors declare that they have no conflict of interest.
